# The Link Between Spontaneous Abortion and Vitamin E may be Influenced by the Fat Mass and Obesity‐Associated Gene, a Case‐Control Study

**DOI:** 10.1002/hsr2.71147

**Published:** 2025-11-27

**Authors:** Zahra Saeedirad, Mahdi Mousavi Mele, Ali Shamsi‐Goushki, Shiva Khodarahmi, Masoomeh Alsadat Mirshafaei, Atefeh Tahavorgar, Sheyda Nami, Seyyed Ali Namakian Yazdi, Niloufar Pourmalek Lahiji, Khadijeh Abbasi Mobarakeh, Sara Khoshdooz, Saeid Doaei, Marjan Ajami, Maryam Gholamalizadeh

**Affiliations:** ^1^ Department of Clinical Nutrition and Dietetics, Faculty of Nutrition and Food Technology Shahid Beheshti University of Medical Sciences Tehran Iran; ^2^ Department of Nutrition Tabriz University of Medical Sciences Tabriz Iran; ^3^ Department of Nutrition, School of Medicine Mashhad University of Medical Sciences Mashhad Iran; ^4^ Shahid Beheshti University of Medical Sciences Tehran Iran; ^5^ Mother and Child Research Center Hamadan University of Medical Sciences Hamadan Iran; ^6^ Department of Physical Education and Sport Sciences Tonekabon Branch, Islamic Azad University Tonekabon Iran; ^7^ School of Nutritional Sciences and Dietetics Tehran University of Medical Sciences Tehran Iran; ^8^ Department of Clinical Biochemistry Iran University of Medical Sciences Tehran Iran; ^9^ Health Sciences in Nutrition, Medical Sciences and Technologies Islamic Azad University Tehran Iran; ^10^ Faculty of Sciences Guilan University of Medical Sciences Rasht Iran; ^11^ Department of Community Nutrition, Nutrition and Food Security Research Center, School of Nutrition and Food Science Isfahan University of Medical Sciences Isfahan Iran; ^12^ Department of Medicine Guilan University of Medical Sciences Rasht Iran; ^13^ Department of Community Nutrition, School of Nutrition and Food Sciences Shahid Beheshti University of Medical Sciences Tehran Iran; ^14^ Reproductive Health Research Center, Department of Obstetrics & Gynecology, Al‐Zahra Hospital, School of Medicine Guilan University of Medical Sciences Rasht Iran; ^15^ Department of Food and Nutrition Policy and Planning Research, National Nutrition and Food Technology Research Institute Shahid Beheshti University of Medical Sciences Tehran Iran; ^16^ Unit of Nutrition and Cancer, Cancer Epidemiology Research Program, Catalan Institute of Oncology Bellvitge Biomedical Research Institute (IDIBELL), L'Hospitalet de Llobregat Barcelona Spain; ^17^ National Nutrition and Food Technology Research Institute, Faculty of Nutrition Sciences and Food Technology Shahid Beheshti University of Medical Sciences Tehran Iran

**Keywords:** alpha‐tocopherol, FTO gene, spontaneous abortion, vitamin E, women's health

## Abstract

**Background and Aims:**

Spontaneous abortion (SA) may be affected by several variables including genetics and lifestyle factors. A recent study discovered that variations in the FTO gene have been associated to both SA and dietary intake. The objective of the study is to examine the association between vitamin E and abortion in individuals with different FTO genotypes.

**Methods:**

This case‐control study was conducted on 539 adult women including 192 women between 20 and 40 years with a history of SA and 347 women without a history of abortion in Tehran, Iran. The blood sample was collected from all participants to evaluate the FTO genotype for rs9939609 polymorphism. A validated Food Frequency Questionnaire was used to assess the intake of vitamin E.

**Results:**

The patients with SA had a lower intake of vitamin E compared to the control group in carriers of the AA genotype of the FTO gene (15.06 ± 7.77 vs. 18.04 ± 12.8 mg/day, *p* = 0.02). SA was inversely associated with dietary intake of vitamin E (*β* = −0.232, *p* = 0.017) and alpha‐tocopherol (*β* = −0.287, *p* = 0.036) only among the cases with AA and AT genotypes of the FTO gene. Adjustments for age, BMI, physical activity, smoking, alcohol drinking, and calorie intake did not change the results.

**Conclusion:**

This study presents the first evidence indicating a significant negative association between SA and dietary intake of vitamin E, specifically among those who have the A allele of the FTO rs9939609 polymorphism.

## 1 Introduction

Spontaneous abortion (SA) refers to the spontaneous or unintentional loss of a fetus, occurring before its development and typically within the first 20 weeks of pregnancy [1]. SA is one of the prevalent negative reproductive outcomes among women around the world, which is a great challenge faced by maternal health promotion [2]. The reported global rate of abortion is estimated to be between 10% and 20% of pregnancies [3, 4]. According to the World Health Organization (WHO), the primary consequences that often occur after a miscarriage include infections, hemorrhage, embolism, and physical health complications [5].

Genetics and lifestyle are important factors associated with the risk of developing SA. The fat mass and obesity‐associated (FTO) gene, which is located on chromosome 16 [6], is responsible for producing a nucleic acid demethylase enzyme that utilizes 2‐oxoglutarate and Fe(II) to catalyze the demethylation of 3‐methylthymine in single‐stranded DNA. This process results in the production of succinate, formaldehyde, and carbon dioxide [7]. FTO mutations result in phenotypic alterations such as the development of numerous congenital malformations [8]. A recent study on the relation between the FTO genotype and recurrent miscarriage found that the FTO gene variant is associated with recurrent miscarriage [9].

Studies have shown that a number of lifestyle factors such as diet, maternal smoking, and alcohol consumption, as well as other factors like obesity, maternal age of 35 years or older, a history of miscarriage or ectopic pregnancy, anemia, a prior urinary tract infection, high blood pressure during pregnancy, and paternal age, all affect the risk of SA [10, 11, 12, 13, 14]. Interestingly, gene variations may influence the link between SA and lifestyle. For example, a recent study found that β‐cryptoxanthin intake was significantly negatively associated with reduced SA in carriers of the TT genotype of the FTO rs9939609 polymorphism (*β* = −0.28, *p* = 0) [15]. Also, an association was found between the FTO genotype and dietary intake of vitamin E [16]. In contrast, no association was observed between SA and other carotenoids in carriers of different FTO genotypes. On the other hand, elevated concentrations of oxidative stress agents could potentially serve as a risk factor for several complications associated with pregnancy such as early SA [17]. A multitude of physiological, pathological, and clinical conditions including pregnancy and its consequences have been associated with oxidative stress and lipid peroxidation [18, 19]. High levels of lipid peroxidation and lower levels of plasma vitamin E were found in women who had abortions, according to these findings [20]. An additional investigation focusing on females undergoing abortions observed that serum lipid peroxide levels peaked just before the initiation of the procedure and decreased considerably subsequently [21].

SA is a multifaceted disorder in which a considerable number of factors appear to be influencing. So far, the interaction between genetic factors and nutritional factors in the occurrence of abortion has been less studied. So, this study is designed to investigate the role of vitamin E in abortion in people with different FTO genotypes. This study aims to reveal that variations in the FTO gene, specifically the rs9939609 polymorphism, may be associated with SA and dietary vitamin E intake and highlights that weather higher consumption of vitamin E is inversely related to SA among women with specific FTO genotypes.

## 2 Methods

This case‐control study was conducted on 539 adult women including 192 women with a history of SA and 347 women without a history of abortion in Tehran, Iran. Participants were randomly selected from individuals visiting Shohadaye Tajrish Hospital for general check‐ups. The inclusion criteria for the case group included a history of at least one miscarriage before the 20th week of pregnancy and the age between 20 and 40 years. The inclusion criteria for the control group included no history of SA and age between 20 and 40 years. Individuals who were taking vitamin E supplements and had no interest to continue participating in the study (due to time constraints and lack of interest) or were unable to provide the necessary information (due to lack of understanding of the questions), were excluded from the study. At the beginning of the study, the purpose and implementation of the study were explained, and written consent was obtained from all participants.

Demographic information such as age and education level, as well as medical history including reproductive system illnesses, diabetes, and hypertension, were gathered using a comprehensive questionnaire and in‐person interviews. Height was quantified in centimeters (cm) with a precision of 0.5 cm, while weight was determined using a digital scale with a precision of 0.5 kg.

### 2.1 FTO Gene Genotyping

The FTO rs9939609 polymorphism was specifically chosen for this study due to its established association with obesity and pregnancy outcomes. Additionally, the prevalence of rs9939609 polymorphism in various populations allows for a broader understanding of its impact on weight‐related phenotypes, making it a valuable marker for investigating the genetic underpinnings of obesity and related health conditions. To evaluate the FTO gene genotype for the presence of rs9939609 polymorphism, 5 cc of blood sample was collected from all participants. Then, using the centrifugation method, blood cells were separated, and DNA was extracted using a standard kit. Master Mix DNA Polymerase (cat. No. A180301; Ampliqon, Denmark) was utilized to amplify the extracted DNA samples via PCR. The PCR products were subsequently subjected to analysis via the tetra‐primer amplification refractory mutation system‐polymerase chain (ARMS‐PCR) to determine the presence of the intended rs9939609 polymorphism.

### 2.2 Dietary Intake

To accurately assess the dietary intake of vitamin E among participants, we employed a validated semi‐quantitative Food Frequency Questionnaire (FFQ). This tool, designed to gather detailed dietary information, allows for the estimation of vitamin E consumption based on frequency and quantity of food items consumed that are known sources of this nutrient. The collected information was converted to daily nutrient intake using Nutritionist IV software.

### 2.3 Statistical Analysis

The normality of the data was assessed using the Kolmogorov‐Smirnov test. The chi‐square test was used to compare the qualitative characteristics of the groups, while the independent *t*‐test was employed to examine the quantitative variables. To examine the impact of the FTO gene genotype on the association between SA and dietary intake of vitamin E, several linear regression models were conducted independently for people with different FTO genotypes, using the dominant genetic model (AA/AT vs. TT). The analyses were conducted using SPSS version 27, with a level of significance set at *p* < 0.05.

### 2.4 Ethics Statement Approval and Consent to Participate

All methods were carried out in accordance with relevant guidelines and regulations Ethical Committee of the Guilan University of Medical Sciences. (code: IR.SBMU.CRC.REC.1403.004). All procedures of the studies involving human participants were by the ethical standards of the institutional and/or national research committee and the 1964 Declaration of Helsinki and its later amendments or comparable ethical standards. All participants signed informed consent forms at the baseline the study.

## 3 Results

Table 1 displays the characteristics of the cases and controls. There are no significant differences between the two groups regarding maternal age, body mass index (BMI), right DBP, and right SBP.

**Table 1:** General characteristics of the participants.

	Cases (*n* = 192)	Controls (*n* = 347)	*p*
Age (Year)	35.84 ± 10.16	32.60 ± 12.86	0.07
Height (Cm)	156.87 ± 6.19	156.80 ± 5.68	0.88
Weight (Kg)	71.89 ± 10.51	70.35 ± 10.29	0.10
BMI (Kg/m^2^)	29.16 ± 4.01	28.59 ± 3.96	0.11
Right DBP (mmHg)	70.52 ± 9.33	70.34 ± 8.80	0.85
Right SBP (mmHg)	109.29 ± 14.03	108.75 ± 14.19	0.73
BUN (mg/dL)	12.50 ± 3.17	12.62 ± 3.51	0.74
Creatinine (mg/mL)	0.97 ± 0.11	0.96 ± 0.11	0.72
PCR result (%) TT	94 (36.96)	40 (28.6)	0.33
AA	22 (8.6)	14 (10.0)	
AT	138 (54.1)	86 (61.4)	
Use alcohol (yes, *n*, %)	17 (4.9)	28 (14.6)	0.001
Tobacco (yes, *n*, %)	20 (5.8)	8 (4.2)	0.27
Has diabetes (yes)	81 (23.3)	60 (31.3)	0.03
Has hypertension (yes)	89 (25.6)	52 (27.1)	0.39
Calorie (kcal/day)	2534.83 ± 437.91	2552.82 ± 674.69	0.74
Protein (g/day)	84.71 ± 21.34	83.79 ± 30.31	0.71
Carbohydrates (g/day)	359.44 ± 73.84	363.17 ± 99.81	0.66
Fats (g/day)	92.13 ± 21.74	94.16 ± 31.69	0.44
Vitamin E (mg/day)	16.62 ± 8.26	18.03 ± 9.69	0.12
Alpha_ tocopherol (mg/day)	11.86 ± 5.27	12.46 ± 7.70	0.34

Abbreviations: ALP, alkaline phosphatase; BUN, blood urea nitrogen; HDLc, high‐density lipoprotein cholesterol; LDL.c, low‐density lipoprotein cholesterol; RBC, red blood cell; Right DBP, right diastolic blood pressure; Right SBP, right systolic blood pressure; SGOT, serum glutamic oxaloacetic transaminase; SGPT, serum glutamic pyruvic transaminase; TG, triglyceride; WBC, white blood cell.

Regarding dietary intake, the cases had a lower intake of vitamin E compared to the control group in carriers of the AA genotype of the FTO gene (15.06 ± 7.77 vs. 18.04 ± 12.8 mg/day, *p* = 0.02) (Table 2).

**Table 2:** Dietary intake of the participants considering FTO rs9939609 genotypes.

	TT			AA			AT		
	Cases	Controls	*p*	Cases	Controls	*p*	Cases	Controls	*p*
Calorie (kcal/d)	2551.98 ± 406.06	2564.6 ± 592.36	0.89	2422.01 ± 286.18	2670.9 ± 821.83	0.24	2543.77 ± 451.05	2546.13 ± 701.83	0.97
Protein (g/day)	83.78 ± 17.17	84.17 ± 25.52	0.92	76.26 ± 14.58	88.87 ± 48.22	0.31	83.19 ± 19.53	83.85 ± 33.23	0.86
Carbohydrates (g/day)	361.24 ± 69.30	364.95 ± 100.38	0.82	344.24 ± 63.79	379.29 ± 122.09	0.31	363.18 ± 61.93	362.45±89.15	0.95
Fats (g/day)	93.68 ± 16.39	95.28 ± 29.49	0.71	88.01 ± 11.89	97.49 ± 26.64	0.19	92.82 ± 26.98	93.10 ± 33.55	0.95
Vitamin E (mg/day)	17.54 ± 8.72	20.14 ± 12.16	0.21	15.06 ± 7.77	18.04 ± 12.8	0.02	17.10 ± 9.03	17.75 ± 7.84	0.78
Alpha_ tocopherol (mg/day)	12.32 ± 5.15	13.88 ± 10.66	0.30	12.01 ± 5.13	12.46 ± 8.64	0.85	11.99 ± 5.8	12.45 ± 5.75	0.53

*Note:* Model 1: Adjusted for other carotenoids, Model 2: Further adjusted for age, Model 3: Further adjustment for BMI and physical activity, smoking, and alcohol drinking, Model 4: Further adjusted for calorie intake.

**Figure 1:**
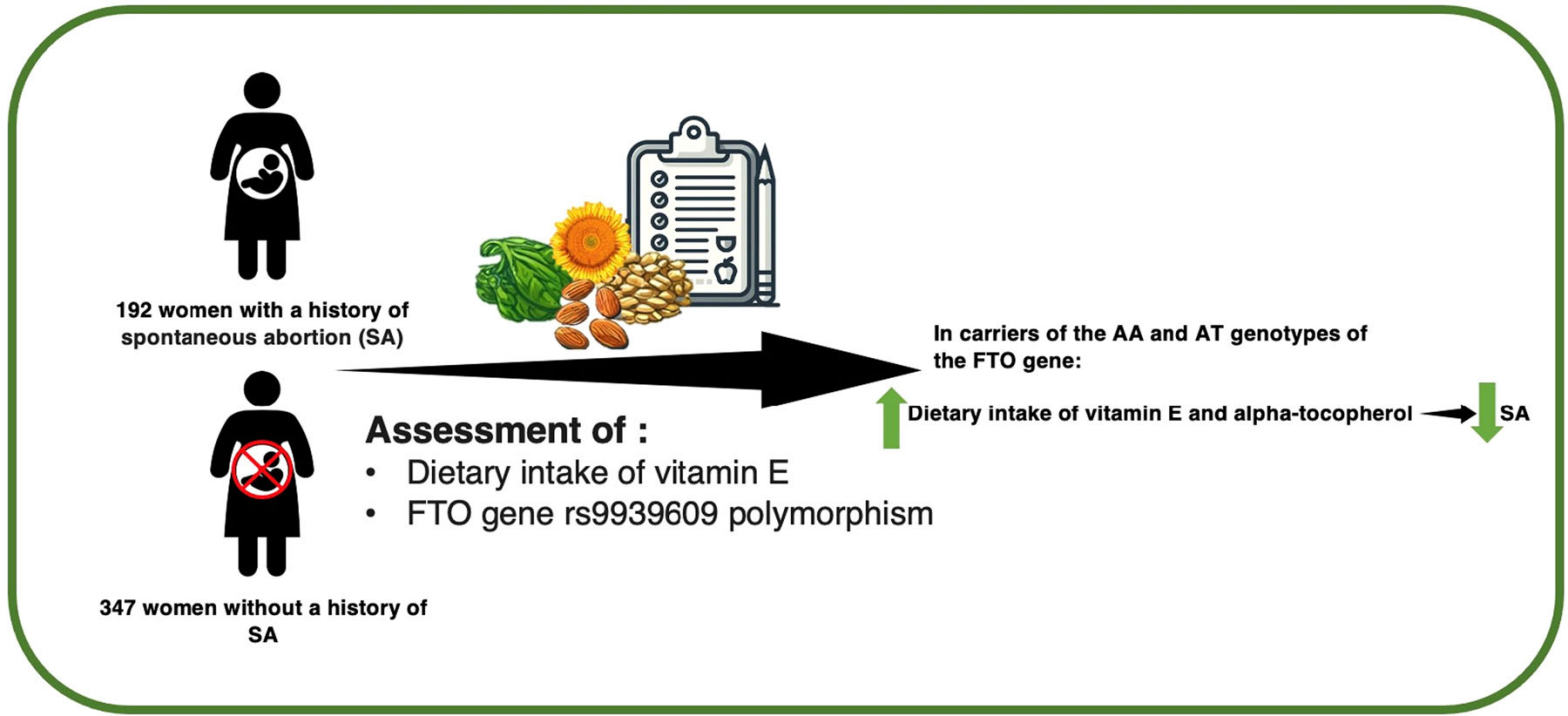
The link between spontaneous abortion and vitamin E and FTO gene.

Regarding the association of SA and dietary intake of vitamin E across carriers of different FTO genotypes, significant inverse associations were found between SA and dietary intake of total vitamin E (*β* = −0.232, *p* = 0.017) and alpha‐tocopherol (*β* = −0.296, *p* = 0.034) after adjustment for another type of vitamin E (Table 3). Further adjustment for age (Model 2), additional adjustment for BMI and physical activity, smoking, and alcohol drinking (Model 3), and further adjustment for calorie intake (Model 4) did not change the results (Table 3) (Figure 1).

**Table 3:** Linear regression of the association between SA and dietary intake of vitamin E considering FTO genotypes.

		Model 1		Model 2		Model 3		Model 4	
		*β*	*p*	*β*	*p*	*β*	*p*	*β*	*p*
TT	Vitamin E	−0.296	0.133	−0.285	0.153	−0.262	0.198	−0.259	0.209
	Alpha‐tocopherol	0.216	0.272	0.204	0.305	0.186	0.363	0.187	0.363
AT	Vitamin E	−0.287	0.036	−0.262	0.045	−0.281	0.041	−0.260	0.048
	Alpha‐tocopherol	−0.254	0.067	−0.033	0.017	−0.015	0.017	−0.033	0.017
AA	Vitamin E	−0.232	0.017	−0.255	0.046	−0.224	0.012	−0.274	0.048
	Alpha‐tocopherol	−0.296	0.034	−0.266	0.012	−0.255	0.020	−0.254	0.248

*Note:* Model 1: Adjusted for another type of vitamin E, Model 2: Further adjusted for age, Model 3: Further adjustment for BMI and physical activity, smoking, and alcohol drinking, Model 4: Further adjusted for calorie intake.

## 4 Discussion

The present study was an attempt to explore and analyze the relationship between vitamin E, abortion, and FTO genotypes. The result indicated that there was a negative association between SA and the intake of vitamin E in individuals with FTO AA and AT genotypes. Adjustments for age, BMI, physical activity, smoking, alcohol drinking, and calorie intake did not change the results.

There are frequent reports of the impact of dietary components on the risk of SA [15, 22]. The findings of this study on the association between vitamin E and SA were consistent with numerous prior studies [23, 24, 25]. Pregnant women are more susceptible to nutritional deficiencies due to the heightened metabolic requirements caused by the developing fetus [26]. During the process of normal placental development, oxidative stress is produced. Numerous observational and interventional studies indicate that nutritional deficiencies in antioxidants, including vitamin E, may present a substantial reproductive risk [27]. Inadequate supply of antioxidants may intensify oxidative stress in both the placenta and maternal circulation, potentially resulting in negative pregnancy outcomes [26].

Vitamin E is an antioxidant that is not produced by enzymes and several studies indicated that pregnant women who consume enough vitamin E are more probable to have a full‐term birth [28]. Additionally, vitamin E has been found to protect and maintain the endometrial membrane from damaging free radicals [29]. Furthermore, fetal death has been related to a low in Vitamin E [27]. It is also shown that reduced levels of vitamin E may cause oxidative stress and recurrent SA in women [23].

The FTO gene was initially identifying through a genome‐wide association study, which identified it as an independent genetic risk factor uniquely contributing to the development of obesity [30]. More recently, it has been shown that the polymorphisms of the FTO gene can contribute to several medical disorders, including cancer, diabetes mellitus, myocardial infarction, and renal failure. It is yet unknown, nevertheless, exactly what function the FTO gene plays in biology [31]. The process of nucleic acid demethylation is mediated by the FTO gene. Given that proper fetal development is predicated on DNA methylation‐based programming, the FTO gene may theoretically serve as a prospective candidate gene associated with susceptibility to SA. Additional evidence for the potential involvement of FTO in fetal development is provided by the observation that FTO is significantly expressed in the placenta of individuals, and that this expression appears to be associated with increased fetal weight and length [32].

This is the first study to our knowledge to examine the impact of the FTO gene rs9939609 polymorphism on the association between SA and vitamin E. The present study exhibited several strengths, including an adequate sample size and thorough adjustments for potential confounding factors, which enhance its credibility and depth of analysis. However, we had several limitations. One limitation of this study is the reliance on self‐reported FFQs, which may introduce recall bias affecting the accuracy of dietary intake data. Also, the lack of causal inference in this case‐control design arises because it only establishes associations between exposure and outcome without determining the temporal sequence or directionality of the relationship. Furthermore, the methodology is inherently prone to certain errors, such as recall bias, which participants may not accurately remember their dietary intake. Moreover, there is a tendency for underreporting of food consumption, particularly among obese and overweight participants.

## 5 Conclusion

In summary, this study provides the first evidence, to our knowledge, on an inverse association between SA and dietary intake of total vitamin E and alpha‐tocopherol in carriers of A allele of FTO rs9939609 polymorphism. If confirmed in future intervention trials, our study may have implications for personalized diets advocating vitamin E and alpha‐tocopherol‐rich diets as a novel preventive approach against SA in genetically susceptible women. Further longitudinal studies are warranted to confirm these results and to discover the underlying mechanisms of the effect of FTO gene polymorphisms on the link between SA and dietary intake of vitamin E. research could benefit from examining a wider array of genetic polymorphisms, as well as conducting RCTs to establish causality and determine effective dietary interventions.

## Author Contributions

**Zahra Saeedirad:** conceptualization. **Mahdi Mousavi Mele:** methodology. **Ali Shamsi‐Goushki:** software. **Shiva Khodarahmi:** supervision. **Masoomeh Alsadat Mirshafaei:** formal analysis. **Atefeh Tahavorgar:** data curation, writing – review and editing. **Sheyda nami:** writing – original draft. **Seyyed Ali Namakian Yazdi:** formal analysis. **Niloufar Pourmalek Lahiji:** software, writing – review andediting. **Khadijeh Abbasi Mobarakeh:** formal analysis. **Sara Khoshdooz:** investigation. **Saeid Doaei:** funding acquisition. **Marjan Ajami:** formal analysis, writing – review and editing. **Maryam Gholamalizadeh:** writing – review and editing.

## Consent

All participants signed informed consent forms.

## Conflicts of Interest

The authors declare no conflicts of interest.

## Transparency Statement

The lead author Saeid Doaei, Marjan Ajami affirms that this manuscript is an honest, accurate, and transparent account of the study being reported; that no important aspects of the study have been omitted; and that any discrepancies from the study as planned (and, if relevant, registered) have been explained.

## Data Availability

The data that support the findings of this study are available from the corresponding author upon reasonable request.
